# What Mistakes Can Be Made When Performing the Electrical Cardioversion Procedure?—Analysis of Emergency Medical Team Performance during the Championships in Emergency Medicine

**DOI:** 10.3390/healthcare12171724

**Published:** 2024-08-29

**Authors:** Michał Ćwiertnia, Mieczysław Dutka, Piotr Białoń, Michał Szlagor, Arkadiusz Stasicki, Monika Mikulska, Maciej B. Hajduga, Rafał Bobiński, Marek Kawecki, Tomasz Ilczak

**Affiliations:** 1 Department of Emergency Medicine, Faculty of Health Sciences, University of Bielsko-Biala, Willowa 2, 43-309 Bielsko-Biała, Poland; pbialon@ubb.edu.pl (P.B.); mszlagor@ubb.edu.pl (M.S.); astasicki@ubb.edu.pl (A.S.); mmikulska@ubb.edu.pl (M.M.); mkawecki@ubb.edu.pl (M.K.); tilczak@ubb.edu.pl (T.I.); 2 European Pre-Hospital Research Network, Nottingham NG11 8NS, UK; 3 Department of Biochemistry and Molecular Biology, Faculty of Health Sciences, University of Bielsko-Biala, Willowa 2, 43-309 Bielsko-Biała, Poland; mdutka@ubb.edu.pl (M.D.); mbhajduga@ubb.edu.pl (M.B.H.); rbobinski@ubb.edu.pl (R.B.)

**Keywords:** cardiopulmonary resuscitation, electric cardioversion, advanced life support

## Abstract

Background: Medical personnel carrying out electrical cardioversion (EC) procedures must remember to have the R-wave sync mode switched on, use the correct energy and maintain personal safety. The defibrillators used by medical response teams most often switch out of cardioversion mode once a shock is delivered. Therefore, this mode must be switched on again before subsequent shocks are delivered. The main aim of the study was to assess the ability of emergency medical teams participating in emergency medicine championships to perform EC. Methods: The research was a retrospective observational study and was based on an analysis of the evaluation sheets from two tasks simulating the management of a patient with unstable tachycardia conducted during the International Winter Emergency Medicine Championships. Three-person teams consisting of paramedics and representing the Polish emergency services were included in the study. The team representing the championship organiser and the few foreign teams participating in the competition were excluded from the study. Results: The decision to conduct EC was taken by 36 teams (83.72%) in 2015 and 27 teams (87.10%) in 2019. In both editions of the championships, during consecutive shocks, the percentage of actions performed correctly decreased significantly—switching on synchronisation mode in 2015 (94.4%, 83.33%, 72.22%) and in 2019 (100%, 88.89%, 81.48%); correct energies in 2015 (91.67%, 80.56%, 77.78%) and in 2019 (92.59%, 85.19%, 81.48%); shocks in a safe manner in 2015 (94.44%, 94.44%, 91.67%) and in 2019 (100%, 96.30%, 96.30%). Conclusions: Teams participating in the assessed tasks in a significant majority of cases correctly qualified the patient for EC, and correctly carried out the actions required for this procedure. It is of particular note that with every subsequent shock, the percentage of shocks carried out without the sync mode increased significantly.

## 1. Introduction

Electrotherapy is one of the most important elements in treating patients in a life-threatening condition. The procedures connected to electrotherapy conducted by members of emergency medical response teams (MRTs) include defibrillation, transcutaneous pacing and EC [1,2]. The EC procedure is the key element in the treatment of patients with unstable tachycardia [3,4]. Correctly conducting this procedure involves ensuring personal safety, using the correct energies and enabling the R-wave sync mode. Maintaining safety is ensured by removing open sources of oxygen to a safe distance, as well as ensuring that nobody touches the patient. The appropriate shock energy depends on the type of heart rhythm and on the number of shocks delivered. EC conducted without sync mode bears the risk of delivering a shock during the relative refractory period of the heart and inducing ventricular fibrillation (VF) [3,4,5,6,7].

The decision to perform EC should always be preceded by a thorough assessment of the patient’s condition. This is due to the differences in the management to be undertaken when the patient is or is not showing signs of haemodynamic instability [3,4]. According to European Resuscitation Council (ERC) guidelines, haemodynamic instability can be indicated by symptoms such as shock, syncope, myocardial ischaemia and severe heart failure. In patients with these symptoms, immediate EC is recommended [3].

Even a fully correct EC procedure carries the risk of causing a sudden cardiac arrest in the patient [4,8]. For this reason, the manufacturers of defibrillators usually programme their devices so that the sync mode automatically switches off after EC is carried out [9]. This makes it possible to immediately carry out defibrillation if necessary [7,9]. This situation requires the medical personnel to switch on sync mode again before every subsequent EC attempt [9].

Improving the knowledge and skills of MRTs can be achieved through participation in emergency medicine championships. Thanks to this type of event, the participants can practise treating patients in a variety of life-threatening conditions, and the organisers can demonstrate what the most common mistakes made by team members are [10]. Since 2006, the Bielsko Emergency Services in Poland have organised the International Winter Championships in Emergency Medicine. Three-person medical response teams from Poland take part in the championships, as well as guest teams invited from abroad.

The main aim of the study was to assess the ability of emergency medical teams participating in the International Winter Emergency Medicine Championships to perform EC. The authors assessed the teams’ ability to recognise the indications for this procedure, the correctness of its performance and the subsequent management when it was found to be ineffective.

## 2. Materials and Methods

This research was a retrospective observational study and was conducted on the basis of detailed analysis of assessment cards for tasks carried out during the International Winter Championships in Emergency Medicine in the years 2013–2023. In these years, tasks related to cardiopulmonary resuscitation were prepared and carried out by European Resuscitation Council (ERC) advanced life support instructors. During this period, in 2015 and 2019, there were two tasks involving simulated treatment of a patient with unstable tachycardia. Three-person teams consisting of paramedics and representing the Polish emergency services from all over the country participated in the championships. All of these teams were included in the study. Of the Polish teams, only the organiser’s team, which was not included in the championship’s generic classification, was excluded from the study. Foreign teams were also excluded from the study, as only a total of three such three-person teams participated in the championships in 2015 and 2019. Teams taking part in the championships were previously registered by the directors of individual units. According to the regulations, only persons currently employed in a given unit could participate. The people preparing and conducting the tasks were in no way connected to any of the championship participants.

The tasks analysed consisted of a ten-minute simulated scenario with an adult with unstable tachycardia. In 2015, the scenario involved a patient with a history of paroxysmal atrial fibrillation, whose symptoms suddenly appeared immediately before the team was called. This patient was unconscious with shock symptoms and features of heart failure. In the 2019 scenario, the same symptoms indicative of hemodynamic instability were present in a patient with ventricular tachycardia.

The procedures were carried out on a MegaCode Kelly mannequin from the firm Laerdal. The assessment cards used by the judges were constructed so as to assess the compliance of the procedures with the then ERC guidelines. Information about the fact that tasks were to be assessed according to ERC guidelines was included in the official championships regulations and participants were also reminded of this before the tasks began. In the analysed tasks, the judges assessed: the decision of the necessity to conduct EC, the enabling of sync mode before every shock, the shock energies, the size of the electrodes/paddles used, personal safety during the shocks, and the administration of amiodarone after the third ineffective shock—the dose, dilution, route and time of administration.

Among the teams who took the decision regarding the necessity of conducting EC, the judges assessed whether the defibrillator was in sync mode before every subsequent shock—in both editions of the championships, all teams used biphasic devices in which this mode was automatically disabled after a shock was delivered. The assessment of the shock energies was based on the then ERC guidelines, as well as on scientific research that the guidelines referred to. In the task, in both 2015 and 2019, the same values were adopted as correct for subsequent shocks. The first shock should have been delivered with an energy of 120–150 J, the second with a higher energy (higher than the first, but not the maximum), and the third on the maximum energy of a given device. Team members should have used paddles/electrodes dedicated for adults. With every shock, it was noted whether any members of the team were touching the patient, and whether open sources of oxygen were removed to a safe distance. After the third ineffective shock, the teams should have begun to administer 300 mg of amiodarone intravenously/intraosseously diluted in 5% glucose within 10–20 min.

For the statistical analysis of the results, the adopted level of significance was *p* = 0.05. In order to test for possible significant differences between the activities covered in the study areas performed at subsequent shocks (enabling sync mode for subsequent shocks, the use of the correct energies for subsequent shocks and the delivery of subsequent shocks in a safe manner), the non-parametric Friedman ANOVA test was used. To precisely determine between which shocks there were significant differences in the individual activities studied, a Bonferroni post hoc test was performed—pairwise comparison. The calculations were made in the R statistical environment version 3.6.0, PSPP version 2.0.0 software and MS Office 2019.

## 3. Results

The research carried out showed that a total of 74 three-person teams representing Polish emergency services took part in both editions of the championship (43 teams in 2015 and 31 teams in 2019).

Table 1 presents the number and percentage of teams that carried out individual actions correctly in the years 2015 and 2019.

Table 2 below present difference between the percentage of enabling sync mode for subsequent shocks.

**Figure 1:**
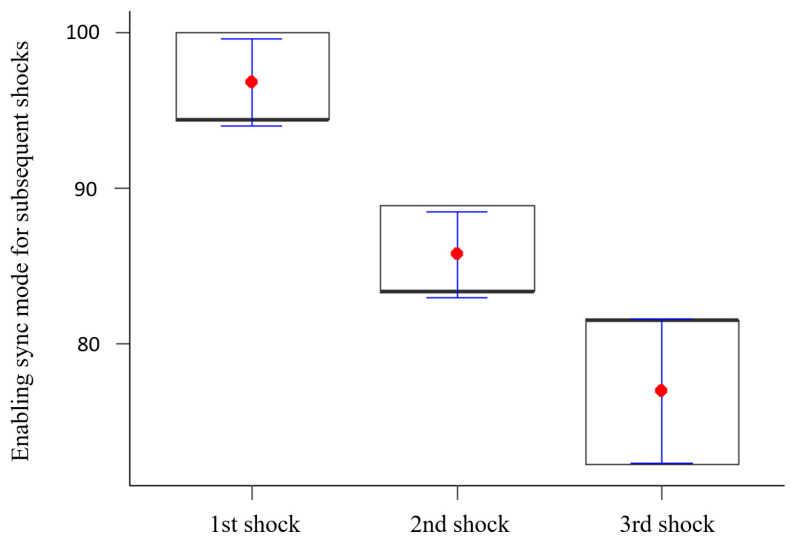
Enabling sync mode for subsequent shocks.

The research showed statistically significant differences between the percentage of enabling sync mode for subsequent shocks (*p* < 0.05). It was shown that the correctness of enabling sync mode was decreased significantly with each successive shock. The results are illustrated in Figure 1.

Table 3 below presents the difference between the percentage of use of the correct energies for subsequent shocks.

**Figure 2:**
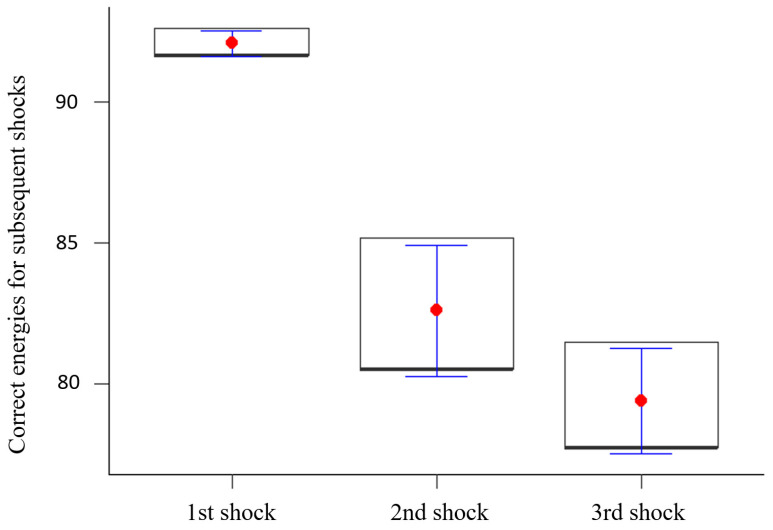
Correct energies for subsequent shocks.

The research showed statistically significant differences between the percentage of use of the correct energies for subsequent shocks (*p* < 0.05). It was shown that the use of the correct energy decreased significantly with each successive shock. The results are illustrated in Figure 2.

Table 4 below present difference between the percentage of delivering subsequent shocks in a safe manner.

**Figure 3:**
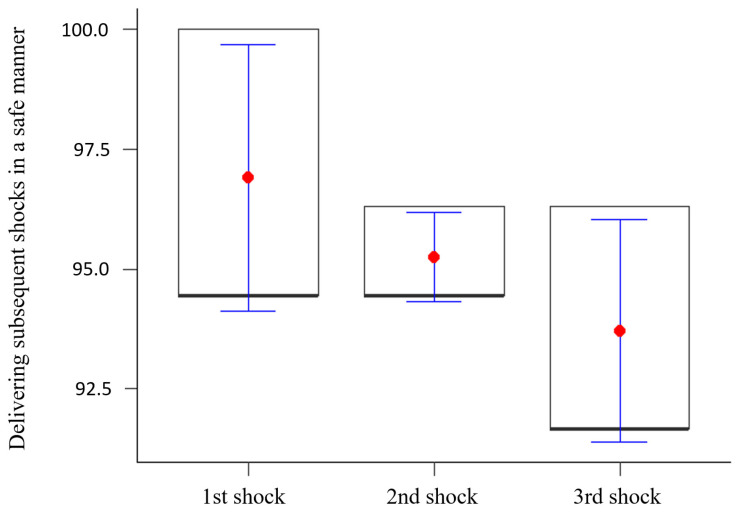
Delivering subsequent shocks in a safe manner.

The research showed a statistically significant difference between the percentage of delivering subsequent shocks in a safe manner. (*p* < 0.05). It was shown that delivering subsequent shocks in a safe manner decreased significantly with each successive shock. The results are illustrated in Figure 3.

## 4. Discussion

In patients with unstable tachycardia, it is necessary to implement the appropriate means of treatment. According to ERC guidelines, this involves immediate use of EC [3]. Our research has shown that, based on the patient’s condition, the majority of the teams assessed took the correct decision on the necessity of conducting EC. In 2015, such a decision was taken by 83.72% of the teams, and in 2019 by 87.10%. In one study assessing the skills of 136 paramedics in performing EC, similar results were obtained. The study showed that the correct decision to perform electrical cardioversion was made in 88.7% of cases [11]. Numerous scientific studies have shown [12,13,14,15] that this type of management in a patient with unstable tachycardia results in a high probability of a quick return to sinus rhythm.

Correct implementation of EC should be synchronised with the R waves. For this reason, members of MRTs should ensure that the sync mode is enabled before every subsequent shock [3,9,16]. Our research has demonstrated that with subsequent shocks there was a significant increase in the number of situations where synchronisation was not enabled. This situation was most certainly directly influenced by the fact that all teams participating in the championships used devices in which the sync mode was automatically disabled after a shock was delivered. Al Duhailib et al. [9] describe how the automatic disabling of the sync mode makes it possible to immediately conduct defibrillation if the shock induces VF. Our research has shown, however, that this can increase the risk that for subsequent shocks MRT members can forget to switch this mode back on again. Situations in which medical personnel, while treating a patient with tachycardia, forgot to turn on synchronisation mode again after an earlier shock are described in the literature [17]. Such procedural errors can lead to a shock being delivered during the heart’s relative refractory period and induce VF [3,18].

According to ERC guidelines, the shock energies for EC should be dependent on the type of heart rhythm and on which subsequent shock is being delivered [1,3]. According to the guidelines [19], in both tasks teams should have delivered the first shock at an energy in the range between 120 and 150 J, and the second shock energy should have been increased so that the third shock was delivered at the maximum energy. Our research has shown that a considerable majority of the teams in both editions of the championships delivered shocks at the correct energies. One survey, conducted in Poland in the form of a questionnaire among paramedics and nurses working in emergency medical teams, showed significantly worse results in terms of knowledge of the EC procedure. These studies showed that only 43.79% of the participants correctly indicated what the shock energy should be during the first EC attempt [20]. In our research, the percentage of correct energies during the first EC attempt was 91.67% in 2015 and 92.59% in 2019. However, it should be noted that during the championship team members had the opportunity to consult with each other before performing individual activities which may have made it easier for them to choose the correct energy. According to some studies [21,22], using the correct shock energies during the EC procedure increases the chances of restoring sinus rhythm.

Shocks delivered during EC should be carried out with the use of paddles or electrodes of an appropriate size [23,24,25]. Paddles or electrodes intended for paediatric patients should not be used for this purpose for adults [26,27]. This research has shown that the teams always used the correct size of paddles/electrodes in both the analysed tasks. As demonstrated in research papers [26,28], the use of the correct size of paddles or electrodes in this way reduces chest impedance, and therefore increases the chances of delivering an effective shock.

A key element of conducting EC is ensuring the personal safety of the team members [3,4]. Studies have described cases in which patients [29,30] or medical personnel [29,31] have sustained serious injury during electrical shocks. Soar et al. [3] describe how during shocks care must be taken to ensure that no-one is touching the patient and that any oxygen is removed to a safe distance. Our research has shown that teams delivered shocks without due regard for safety in only a minimal percentage of cases. According to the remarks entered by the judges onto assessment cards, these cases mainly amounted to a failure to move an oxygen source to a safe distance. Such behaviour can result in the patient and team members suffering burns [29,31]. A system of regular training can be helpful in eliminating such significant errors. As scientific studies have shown, a short time after training, medical personnel lose knowledge [32,33] and confidence [34] in performing the EC procedure. This situation may also be influenced by the fact that some medical personnel, including EMS personnel, perform this procedure quite infrequently. In their study, Sokolowski et al. [20] showed that as many as 65.09% of the paramedics and nurses working in the EMSs in southwestern Poland had never performed EC. Therefore, systematic participation in training courses on EC [33,34] and especially those in which the use of manual defibrillators is discussed in detail [32] can significantly reduce the number of errors that occur when performing this procedure.

If three attempts at EC on a patient with unstable tachycardia turn out to be ineffective, 300 mg of amiodarone diluted in 5% glucose should be administered intravenously or intraosseously within 10–20 min [3]. Our research has shown that the decision to administer amiodarone was taken by 83.33% of the teams in 2015 and 83.87% of the teams in 2019. These teams always administered the medicine using the correct route and in the vast majority of cases in the correct dose and correct dilution. Another study assessing the knowledge of EMS personnel showed that only 66.86% of them could indicate the correct dose of amiodarone to be given in this situation [20]. In the case of our own study, the correct dose of amiodarone was given by 93.33% of the teams in 2015 and 95.65% in 2019. The implementation by the teams of pharmacological cardioversion in this way is a procedure recommended by the ERC for patients with unstable tachycardia in situations when EC is shown to be ineffective [3]. 

The results of our study, combined with those of other authors [11,17,20,32,33,34], may indicate a real existing problem associated with performing the EC procedure. Therefore, special emphasis should be placed on the proper education of paramedics and then systematically training those already working in EMS. As authors, we can propose the introduction into the protocol and recommendations of scientific societies of the principle of confirmation in the form: “Say it loud and say it twice”—where each member of the team checks and confirms the fact that the correct energy is set, synchronisation is turned on and safety is maintained.

The authors of the study are aware of some of its limitations. First of all, the results obtained from this study come from a time when ERC guidelines for the treatment of patients with unstable atrial fibrillation differed from those currently recommended. Another limitation is that the paper does not present descriptive statistics of the study group, as such information was not collected by the championship organisers. The study was conducted in a group of paramedics, so in the future it is necessary to expand it to include a group of doctors and nurses. A limitation of the study is probably also the fact that the participants of the championship were probably chosen as representatives of their EMS units to obtain the best possible result, so they may have had above-average knowledge and skills.

## 5. Conclusions

Our research based on simulated scenarios has shown that the teams in most cases used the correct management method for selecting energy, enabling the synchronisation mode and maintaining safety when performing the EC procedure. A way to eliminate errors that occur in this area can be systematic training, directed especially to paramedics who rarely perform this procedure. Particularly noteworthy is the fact that with every subsequent shock, there was a significant increase in the percentage of cases of shocks delivered without the use of sync mode. As a result, the study authors suggest that it should be considered that defibrillators be programmed so that the devices remain in sync mode after shocks are delivered. However, this situation requires additional research to assess the effect of sync mode not being disabled on the time when defibrillation can be conducted if EC induces VF.

## Figures and Tables

**Table 1:** Studied actions carried out correctly by MRT members in the years 2015 and 2019.

	2015		2019	
	*n*	%	*n*	%
Decision of the necessity to conduct EC	36	83.72	27	87.10
Enabling cardioversion mode before 1st shock	34	94.44	27	100
Enabling cardioversion mode before 2nd shock	30	83.33	24	88.89
Enabling cardioversion mode before 3rd shock	26	72.22	22	81.48
1st shock energy	33	91.67	25	92.59
2nd shock energy	29	80.56	23	85.19
3rd shock energy	28	77.78	22	81.48
Size of paddles/electrodes during 1st shock	36	100	27	100
Size of paddles/electrodes during 2nd shock	36	100	27	100
Size of paddles/electrodes during 3rd shock	36	100	27	100
Personal safety during 1st shock	34	94.44	27	100
Personal safety during 2nd shock	34	94.44	26	96.30
Personal safety during 3rd shock	33	91.67	26	96.30
Decision to administer amiodarone	30	83.33	23	83.87
Amiodarone dose	28	93.33	22	95.65
Amiodarone administering route	30	100	23	100
Time of administering amiodarone	26	86.67	21	91.30
Amiodarone dilution	29	96.67	23	100

*n*—number of teams.

**Table 2:** Difference between the percentage of enabling sync mode for subsequent shocks.

	χ^2^	df	*p*	M	SD	Min	Max	Me
1st shock	98.00	2	<0.001	96.82	2.77	94.44	100.00	94.44
2nd shock				85.74	2.78	83.33	88.89	83.33
3rd shock				76.94	4.67	72.22	81.48	81.48

χ^2^—test statistic; df—degrees of freedom; *p*—statistical significance; M—mean; SD—standard deviation; Min—minimum; Max—maximum, Me—median.

**Table 3:** Difference between the percentage of use of the correct energies for subsequent shocks.

	χ^2^	df	*p*	M	SD	Min	Max	Me
1st shock	100.00	2	<0.001	92.07	0.46	91.67	92.59	91.67
2nd shock				82.61	2.32	80.56	85.19	80.56
3rd shock				79.41	1.86	77.78	81.48	77.78

χ^2^—test statistic; df—degrees of freedom; *p*—statistical significance; M—mean; SD—standard deviation; Min—minimum; Max—maximum; Me—median.

**Table 4:** Difference between the percentage of delivering subsequent shocks in a safe manner.

	χ^2^	df	*p*	M	SD	Min	Max	Me
1st shock	88.92	2	<0.001	96.90	2.79	94.44	100.00	94.44
2nd shock				95.25	0.93	94.44	96.30	94.44
3rd shock				93.71	2.32	91.67	96.30	91.67

χ^2^—test statistic; df—degrees of freedom; *p*—statistical significance; M—mean; SD—standard deviation; Min—minimum; Max—maximum; Me—median.

## Data Availability

The datasets used and/or analysed during the current study are available from the corresponding author on reasonable request.

## References

[1] Perkins G.D., Grasner J.T., Semeraro F., Olasveengen T., Soar J., Lott C., Van de Voorde P., Madar J., Zideman D., Mentzelopoulos S. (2021). European Resuscitation Council Guidelines 2021: Executive summary. Resuscitation.

[2] Merchant R.M., Topjian A.A., Panchal A.R., Cheng A., Aziz K., Berg K.M., Lavonas E.J., Magid D.J. (2020). Part 1: Executive Summary: 2020 American Heart Association Guidelines for Cardiopulmonary Resuscitation and Emergency Cardiovascular Care. Circulation.

[3] Soar J., Böttiger B.W., Carli P., Couper K., Deakin C.D., Djärv T., Lott C., Olasveengen T., Paal P., Pellis T. (2021). European Resuscitation Council Guidelines 2021: Adult advanced life support. Resuscitation.

[4] Panchal A.R., Bartos J.A., Cabañas J.G., Donnino M.W., Drennan I.R., Hirsch K.G., Kudenchuk P.J., Kurz M.C., Lavonas E.J., Morley P.T. (2020). Part 3: Adult Basic and Advanced Life Support: 2020 American Heart Association Guidelines for Cardiopulmonary Resuscitation and Emergency Cardiovascular Care. Circulation.

[5] Brandes A., Crijns H.J.G.M., Rienstra M., Kirchhof P., Grove E.L., Pedersen K.B., Van Gelder I.C. (2020). Cardioversion of atrial fibrillation and atrial flutter revisited: Current evidence and practical guidance for a common procedure. Europace.

[6] Nguyen S.T., Belley-Côté E.P., Ibrahim O., Um K.J., Lengyel A., Adli T., Qiu Y., Wong M., Sibilio S., Benz A.P. (2023). Techniques improving electrical cardioversion success for patients with atrial fibrillation: A systematic review and meta-analysis. Europace.

[7] Goyal A., Sciammarella J.C., Chhabra L., Singhal M. (2024). Synchronized Electrical Cardioversion. 2023 Mar 27. StatPearls [Internet].

[8] Bonfanti L., Annovi A., Sanchis-Gomar F., Saccenti C., Meschi T., Ticinesi A., Cervellin G. (2019). Effectiveness and safety of electrical cardioversion for acute-onset atrial fibrillation in the emergency department: A real-world 10-year single center experience. Clin. Exp. Emerg. Med..

[9] Al Duhailib Z., Trusz-Gluza M., Jankowski M. Electrical Cardioversion. McMaster Textbook of Internal Medicine. Kraków: Medycyna Praktyczna. https://empendium.com/mcmtextbook/chapter/B31.IV.24.63.

[10] Smart J.R., Kranz K., Carmona F., Lindner T.W., Newton A. (2015). Does real-time objective feedback and competition improve performance and quality in manikin CPR training-a prospective observational study from several European EMS. Scand. J. Trauma Resusc. Emerg. Med..

[11] Hakyemez F., Kara H. (2021). Assesment of the Knowledge and Skills of Paramedics Working in Prehospital Health Services on Making a Decision for and Applying Defibrillation and Cardioversion. Anatol. J. Emerg. Med..

[12] Stiell I.G., Eagles D., Nemnom M.J., Brown E., Taljaard M., Archambault P.M., Birnie D., Borgundvaag B., Clark G., RAFF Investigators (2021). Adverse Events Associated with Electrical Cardioversion in Patients with Acute Atrial Fibrillation and Atrial Flutter. Can. J. Cardiol..

[13] Fried A.M., Strout T.D., Perron A.D. (2021). Electrical cardioversion for atrial fibrillation in the emergency department: A large single-center experience. Am. J. Emerg. Med..

[14] Strzelczyk T.A., Kaplan R.M., Medler M., Knight B.P. (2017). Outcomes associated with electrical cardioversion for atrial fibrillation when performed autonomously by an advanced practice provider. JACC Clin. Electrophysiol..

[15] Prasai P., Shrestha D.B., Saad E., Trongtorsak A., Adhikari A., Gaire S., Oli P.R., Shtembari J., Adhikari P., Sedhai Y.R. (2023). Electric Cardioversion vs. Pharmacological with or without Electric Cardioversion for Stable New-Onset Atrial Fibrillation: A Systematic Review and Meta-Analysis. J. Clin. Med..

[16] Schmidt A.S., Lauridsen K.G., Adelborg K., Torp P., Bach L.F., Jepsen S.M., Hornung N., Deakin C.D., Rickers H., Løfgren B. (2017). Cardioversion Efficacy Using Pulsed Biphasic or Biphasic Truncated Exponential Waveforms: A Randomized Clinical Trial. J. Am. Heart Assoc..

[17] Pchejetski D., Alam T., Alshaker H. (2020). Unsynchronised Cardioversion as a Cause of Ventricular Tachycardia in a Patient with Atrial Fibrillation. Cardiol. Case Rep..

[18] Ikeda S., An Y., Yanagisawa M., Ishigami K., Aono Y., Doi K., Ishii M., Iguchi M., Ogawa H., Masunaga N. (2020). Iatrogenic ventricular fibrillation caused by inappropriately synchronized cardioversion in a patient with pre-excited atrial fibrillation: A case report. J. Cardiol. Cases.

[19] Soar J., Nolan J.P., Böttiger B.W., Perkins G.D., Lott C., Carli P., Pellis T., Sandroni C., Skrifvars M.B., Smith G.B. (2015). European Resuscitation Council Guidelines for Resuscitation 2015: Section 3. Adult advanced life support. Resuscitation.

[20] Sokolowski M., Wawrzynska M. (2019). Knowledge of and attitudes to emergency tachyarrhythmia treatment among paramedics and nurses. Disaster Emerg. Med. J..

[21] Patti L., Ashurst J.V. (2024). Supraventricular Tachycardia. [Updated 2023 Aug 7]. StatPearls [Internet].

[22] Darrat Y., Leung S., Elayi L., Parrott K., Ogunbayo G., Kotter J., Sorrell V., Gupta V., Anaya P., Morales G. (2023). A stepwise external cardioversion protocol for atrial fibrillation to maximize acute success rate. Europace.

[23] Pal-Jakab A., Nagy B., Kiss B., Zima E. (2024). The Influence of Transthoracic Impedance on Electrical Cardioversion and Defibrillation: Current Data [Internet]. Updates on Cardiac Defibrillation, Cardioversion and AED Development.

[24] Vostrikov V.A., Razumov K.V., Gorbunov B.B. (2016). Impact of Electrode Size on the Efficacy of Electrical Cardioversion for Paroxysmal Atrial Fibrillation. Biomed. Eng..

[25] Roh S.Y., Ahn J., Lee K.N., Baek Y.S., Kim D.H., Lee D.I., Shim J., Choi J.I., Kim Y.H. (2021). The Impact of Personal Thoracic Impedance on Electrical Cardioversion in Patients with Atrial Arrhythmias. Medicina.

[26] Van de Voorde P., Turner N.M., Djakow J., de Lucas N., Martinez-Mejias A., Biarent D., Bingham R., Brissaud O., Hoffmann F., Johannesdottir G.B. (2021). European Resuscitation Council Guidelines 2021: Paediatric Life Support. Resuscitation.

[27] Heyer Y., Baumgartner D., Baumgartner C.A. (2022). Systematic Review of the Transthoracic Impedance during Cardiac Defibrillation. Sensors.

[28] Yin R.T., Taylor T.G., de Graaf C., Ekkel M.M., Chapman F.W., Koster R.W. (2023). Automated external defibrillator electrode size and termination of ventricular fibrillation in out-of-hospital cardiac arrest. Resuscitation.

[29] Beebeejaun A., Howard R. (2020). Dangers of defibrillation in flight. J. Australas. Soc. Aerosp. Med..

[30] Koda E.K. (2020). Lumbar Compression Fracture Caused by Cardioversion. Am. J. Case Rep..

[31] Gibbs W., Eisenberg M., Damon S.K. (1990). Dangers of defibrillation: Injuries to emergency personnel during patient resuscitation. Am. J. Emerg. Med..

[32] Siebert J.N., Glangetas A., Grange M., Haddad K., Courvoisier D.S., Lacroix L. (2022). Impact of blended learning on manual defibrillator’s use: A simulation-based randomized trial. Nurs. Crit. Care.

[33] Kowalski C., Boulesteix A.L., Harendza S. (2022). Effective methods to enhance medical students’ cardioversion and transcutaneous cardiac pacing skills retention—A prospective controlled study. BMC Med. Educ..

[34] Smith A.W., Elliott J.O., Gable B.D. (2021). Simulation Improves Internal Medicine Resident Confidence With Defibrillation, Cardioversion, and Transcutaneous Pacemaker Use. Cureus.

